# Supplementary material for: HIV risk behavior and HIV testing among rural and urban men who have sex with men in Zhejiang Province, China: A respondent-driven sampling study
Source: PLoS One. 2020 Apr 2;15(4):e0231026. doi: 10.1371/journal.pone.0231026 (PMC7117739; doi:10.1371/journal.pone.0231026)
Supplement: S1 File — (DOCX) [file pone.0231026.s001.docx]

ID编号：

（粘贴ID编号条码处）

征募卡编号：

**同志人群健康状况调查表**

调查对象姓名/化名:

调查地区：

调查机构：

二〇一三年十一月

**调查对象筛选**

| **S1** | 您的出生日期： 年 月 | | |
| --- | --- | --- | --- |
|  | **S11** | 周岁年龄是否≥14岁？ **①**是 **②**否 **(不纳入调查)** |  |
| **S2** | 您的现住址： 市 县（区），居住时长有 年**（不到一年转换成小数）** | | |
|  | **S21** | 现住址居住时长是否≥3个月？ **①**是 **②**否 **(不纳入调查)** |  |
| **S3** | 过去一年您是否与同性发生过口交或肛交性行为？ **①**是 **②**否 **(不纳入调查)** | |  |

**A. 基本信息**

| **A1** | 婚姻状况：**①**同居**（与女性）** **②**同居**（与男性）** **③**单身 **④**已婚有配偶 **⑤**离异或丧偶 |  |
| --- | --- | --- |
| **A2** | 文化程度：**①**小学及以下 **②**初中 **③**高中/中专 **④**大专及以上 |  |
| **A3** | 目前从事的主要职业：  **①**服务行业职员（餐饮/酒店/美容美发/物业物流等行业） **②**公司职员 **③**经商  **④**政府机关/事业单位员工 **⑤**工人（建筑业/制造业等） **⑥**学生 **⑦**农民  **⑧**离退休人员 **⑨**无业 **⑩**其他（请注明） |  |
| **A4** | 您的月收入是多少元？  **①**无收入 **②**<1000 **③**1000-1999 **④**2000-2999 **⑤**3000-3999 **⑥**≥4000 |  |
| **A5** | 您享受以下哪些医疗保险**（可多选）**？   1. 新型农村合作医疗 **②**城镇职工医保 **③**城镇居民医保 **④**商业保险 **⑤**无 |  |
| **A6** | 您认为自己是属于下列哪种性取向？  **①**同性恋 **②**异性恋 **③**双性恋 **④**未确定 |  |
| **A7** | 您寻找性伴最常去的场所是：  **①**酒吧、歌舞厅、茶室、会所 **②**浴池、桑拿、足疗、按摩 **③**公园、公厕、草地  **④**互联网联系 **⑤**其他（请注明） |  |
| **A8** | 在本地，您大约认识 个圈内的朋友**（男男性行为者）?[认识是指您能够认识对方面孔，知道名字或化名、昵称，和联系方式，并能在一个月内联系上对方。给与调查对象一些时间，仔细回想]** | |
| **A9** | 介绍您来参加这次活动的人和您是什么关系？  **①**过去的性伴**（6个月以前）** **②**近6个月的性伴 **③**好朋友  **④**普通朋友 **⑤**熟人 **⑥**陌生人 |  |
| **A10** | 您感觉目前您的身体健康状况？ **①**很好 **②**好 **③**一般 **④**不好  **⑤**很差 |  |

**B. 性行为特征和性伴网络**

| **B1** | 您**首次**发生**同性**性行为的年龄 ？ | | | | | | | |
| --- | --- | --- | --- | --- | --- | --- | --- | --- |
| **B2** | **最近6个月**，您与 位**同性**性伴发生过**肛交**性行为？**（若无跳至B4）** | | | | | | | |
| **B3** | **最近6个月**，您发生**同性肛交**性行为时安全套使用频率？ | | | | | | | |
|  | **B31** | 固定同性性伴肛交：**①**从未使用 **②**有时使用 **③**每次都用 **④**未发生此类性行为 | | | | | |  |
|  | **B32** | 临时同性性伴肛交**（非商业非固定）**：**①**从未使用 **②**有时使用 **③**每次都用 **④**未发生此类性行为 | | | | | |  |
|  | **B33** | 男性性工作者肛交：**①**从未使用 **②**有时使用 **③**每次都用 **④**未发生此类性行为 | | | | | |  |
|  | **B34** | 群体性肛交： **①**从未使用 **②**有时使用 **③**每次都用 **④**未发生此类性行为 | | | | | |  |
|  | **B35** | 酒后肛交： **①**从未使用 **②**有时使用 **③**每次都用 **④**未发生此类性行为 | | | | | |  |
|  | **B36** | 药物使用后肛交： **①**从未使用 **②**有时使用 **③**每次都用 **④**未发生此类性行为 | | | | | |  |
| **B4** | **最近6个月**，您与 位**同性**性伴发生过**口交**性行为？**（若无跳至B6）** | | | | | | | |
| **B5** | **最近6个月**，您发生**同性口交**性行为时安全套使用频率 | | | | | | | |
|  | **B51** | 固定同性性伴口交： **①**从未使用 **②**有时使用 **③**每次都用 **④**未发生此类性行为 | | | | | |  |
|  | **B52** | 临时同性性伴口交（**非商业非固定**）：**①**从未使用 **②**有时使用 **③**每次都用 **④**未发生此类性行为 | | | | | |  |
|  | **B53** | 男性性工作者口交： **①**从未使用 **②**有时使用 **③**每次都用 **④**未发生此类性行为 | | | | | |  |
| **B6** | 请逐一回忆您在**最近1年**的**同性性伴**，您不需说出他们的名字，但是为了帮助您回忆这些信息，您可以给他们编号或者使用代号（**请按照性行为频次从多到少依次列出**，5人以内，全部列出；超过5人，仅填5人） | | | | | | | |
|  | **性伴特征** | | **1号** | **2号** | **3号** | **4号** | **5号** | |
|  | **B61** | **你们的关系：**  **①**固定同性性伴 **②**临时同性性伴**（非商业非固定）**  **③**商业性同性行为 **④**其他 |  |  |  |  |  | |
|  | **B62** | **性伴年龄：①**<20岁 **②**20～ **③**30～ **④**40～ **⑤**≥50 |  |  |  |  |  | |
|  | **B63** | **性行为频率：**  **①**≥2次/周 **②**4～7次/月 **③**1~3次/月 **④**<1次/月 |  |  |  |  |  | |
|  | **B64** | **性伴HIV感染情况：①**阳性 **②**阴性 **③**不详 |  |  |  |  |  | |
| **B7** | 您**首次**发生**异性**性行为的年龄？**①** 岁 **②**未发生过异性性行为**（跳至C1）** | | | | | | |  |
| **B8** | **最近6个月**，您与 位**异性**性伴发生过性行为？**（若无跳至C1）** | | | | | | | |
| **B9** | **最近6个月**，您发生**异性**性行为时安全套使用频率 | | | | | | | |
|  | **B91** | 配偶**(固定)**异性性伴： **①**从未使用 **②**有时使用 **③**每次都用 **④**未发生此类性行为 | | | | | |  |
|  | **B92** | 临时异性性性伴（**非商业非固定**）：**①**从未使用 **②**有时使用 **③**每次都用 **④**未发生此类性行为 | | | | | |  |
|  | **B93** | 女性性工作者： **①**从未使用 **②**有时使用 **③**每次都用 **④**未发生此类性行为 | | | | | |  |

**C. 行为和社会文化特征**

| **C1** | 您是否吸烟？**[吸烟指每天吸烟1支或以上，且持续一年以上或短期内(≤3月)抽烟达到300支以上]**  **①**现在吸烟 **②**曾经吸烟 **(最近6个月内未曾吸过烟)** **③**从不吸烟 **(跳至D2)** | |  |
| --- | --- | --- | --- |
|  | **D11** | 您从 岁（或 年）开始吸烟？至今，扣除不吸烟的时间，累计吸烟约有 年 | |
|  | **D12** | 平均每天吸 支烟？**(一般一包20支烟)** | |
| **C2** | 您是否饮酒？**[饮酒指每周酒精摄入量达到100克(2两)，如40度酒摄入量为250克，10度酒摄入量为1千克]**  **①**现在饮酒 **②**曾经饮酒**(最近6个月不饮酒)** **③**从不饮酒**(跳至D5)** | |  |
|  | **D21** | 您从 岁（或 年）开始饮酒？至今，扣除不饮酒的时间，累计饮酒约有 年 | |
|  | **D22** | 平均每周饮酒 次，一般每次饮酒量：  **①**啤酒 瓶（ 毫升） **②**红酒 瓶（ 毫升） **③**白酒 两（ 度酒）  **④**黄酒 两（ 度酒） **⑤**其他， 酒**(种类)** 毫升（ 度酒） | |
| **C3** | **最近1年**，您是否有醉酒情况？ **①**经常有 **②**偶尔有 **③**从未有**(跳至D5)** | |  |
| **C4** | **最近1年**，您是否想要或者需要降低您的饮酒量？ **①**是 **②**否 | |  |
| **C5** | **最近1年**，您是否有药物**(包括：精神药品，如镇静催眠药、咖啡因、麦司卡林、LSD，以及麻醉药品，如阿片类、可卡因类、大麻类等)**使用超过本应该的量？   1. 经常有 **②**偶尔有 **③**从未有**(跳至D7)** | |  |
| **C6** | **最近1年**，您是否想要或者需要降低您的药物使用量？ **①**是 **②**否 | |  |
| **C7** | 您是否有过自杀倾向？ **①**从没有过 **②**偶尔有 **③**经常有 | |  |
| **C8** | 您是否遭受过同性性伴暴力？**（性伴暴力包括：身体、性或心理暴力）**   1. 从没有过 **②**偶尔有 **③**经常有 | |  |
| **C9** | 您儿童时期是否遭受性虐待？ **①**是 **②**否 | |  |
| **C10** | 您认为目前社会对同志人群的看法对您的性行为会产生怎样的影响？  **①**增加有保护同性性行为 **②**增加有保护异性性行为 **③**增加无保护同性性行为  **④**增加无保护异性性行为 **⑤**没有影响 | |  |
| **C11** | 您认为您感染HIV的可能性？ **①**很可能 **②**可能 **③**不太可能 **④**不可能 | |  |
| **C12** | 您估计目前同志人群HIV感染率？ **①**≤5% **②**6-10% **③**11-15% **④**16-20% **⑤**≥21% | |  |
| **C13** | 最近一年，您接受艾滋病/性病宣传干预**（包括：安全套/润滑剂发放，性病检查或治疗，艾滋病/性病咨询、宣**  **传材料，预防艾滋病/性病知识培训等）**的频率？   1. 无 **②**1~2次/年 **③**2~5次/半年 **④**1~3次/月 **⑤**1~4次/月 | |  |

**D. 农村MSM**

| **D1** | **最近1年**，您是否在浙江省的城市内发生过同性性行为？ **①**是 **②**否 |  |
| --- | --- | --- |
| **D2** | **最近1年**，您是否在其他省发生过同性性行为？ **①**是 **②**否 |  |

**E. 检测史和检测意愿**

| **E1** | 您以前是否做过HIV检测? **①**是 **②**否**（跳至E6）** | |  |
| --- | --- | --- | --- |
| **E2** | **最近1年**，您HIV检测次数： 次 | | |
| **E3** | 您**最近一次**HIV检测时间： 年 月 | | |
| **E4** | 您**最近一次**HIV检测类型？  **①**自愿咨询检测 **②**疾控系统主动提供的检测服务 **③**输血（血制品） **④**献血 **⑤**就业体检  **⑥**婚前体检 **⑦**住院检查 **⑧**入学体检 **⑨**其他（请注明） | |  |
| **E5** | 您是否有定期检测HIV的习惯？ **①**是 **②**否**（跳至E6）** | |  |
|  | **E51** | 您定期检测都出于哪些原因**（可多选）**？  **①**健康监测 **②**经常发生高危性行为 **③**对固定性伴不信任  **④**固定性伴是阳性 **⑤**不断开始一段新的伴侣关系 **⑥**其他（请注明） |  |
|  | **E52** | 您一般多长时间做一次HIV检测？**（回答完毕跳至F1）**  **①**3个月 **②**半年 **③**一年 **④**其他（请注明） |  |
| **E6** | 您是否愿意接受HIV定期检测？ **①**是**（跳至F1）** **②**否 | |  |
|  | **E61** | 如果您不愿意，原因是**（可多选）**?  **①**不知道去哪里检测 **②**工作或生活习惯问题，很难定期 **③**担心检测结果阳性  **④**检测点不方便 **⑤**害怕暴露自己的身份后受到歧视 **⑥**没有高危行为  **⑦**性伴固定，很安全 **⑧**其他（请注明） |  |

**本次问卷调查到此结束！感谢您的参与！欢迎您对这次调查提出您的宝贵意见！**

**­**

**以下结果由调查人员填写**

| **F1** | 梅毒检测结果：第一种方法：①阳性 ②阴性 |  |
| --- | --- | --- |
| **F2** | 梅毒检测结果：第二种方法：①阳性 ②阴性 |  |
| **HIV既往感染者（不包括本次HIV检测阳性者）仅填写G6——G8** | | |
| **F3** | 本次HIV初筛检测方法： |  |
| **F4** | HIV筛查检测结果： ①阳性 ②阴性**（结束调查）** |  |
| **F5** | HIV确认结果： ①阳性 ②阴性**（结束调查）** |  |
| **F6** | 疫情卡编号： |  |
| **F7** | HIV病毒载量检测结果： ，病毒载量检测日期： 年 月 日 | |
| **F8** | CD4结果： ，CD4检测日期： 年 月 日 | |

**­ ­ ­**

**请确定是否已完成以下流程：①对象筛选 ②知情同意 ③问卷完成后的缺漏项审核**

**调查员： 调查日期： ­ 年 月 日**
