# Supplementary material for: HIV risk behavior and HIV testing among rural and urban men who have sex with men in Zhejiang Province, China: A respondent-driven sampling study
Source: PLoS One. 2020 Apr 2;15(4):e0231026. doi: 10.1371/journal.pone.0231026 (PMC7117739; doi:10.1371/journal.pone.0231026)
Supplement: S2 File — (DOCX) [file pone.0231026.s002.docx]

ID code：

（Paste ID code ）

Coupon code：

**Health questionnaire for men who have sex with men (MSM)**

Name or alia of participant:

Investigated area：

Investigation agency：

November,2013

**Participant inclusion and exclusion**

| **S1** | Birthday：___month ___year | | |
| --- | --- | --- | --- |
|  | **S11** | If age≥14 years old？**①**Yes **②**No**(not included in this survey)** |  |
| **S2** | Recent resident address：___Prefecture(District) ___City，resident time ___year(s)**（less than 1 year is converted into decimal）** | | |
|  | **S21** | If you reside in recent place ≥3 months？**①**Yes **②**No**(not included in this survey)** |  |
| **S3** | If you have oral or anal sex with male sexual partner(s) in the past year **①**Yes **②**No **(not included in this survey)** | |  |

**A. Basic information**

| **A1** | Marital status：①Cohabitation(with female) ②Cohabitation(with male) ③Single ④Married with spouse Divorced or windowed |  |
| --- | --- | --- |
| **A2** | Educational level:  **①**Primary school or below **②**Junior school **③**High school or second school **④**College or above |  |
| **A3** | Occupation：   1. Employee（Restaurant/hotel/beauty salon/property logistics and other industries）**②**Company employees **③**Businessman **④**Government agency/institution staff **⑤**Worker（Construction/manufacturing, etc）**⑥**Student **⑦**Farmer **⑧**Retired **⑨**Unemployed **⑩**Others（note） |  |
| **A4** | Monthly income(RMB: yuan)？   1. No income **②**<1000 **③**1000-1999 **④**2000-2999 **⑤**3000-3999 **⑥**≥4000 |  |
| **A5** | Medical insurance（Single or multiple choices）？  ①[New](javascript:void(0);) [Rural](javascript:void(0);) [Co-operative](javascript:void(0);) [Medical](javascript:void(0);) [System](javascript:void(0);)②The medical insurance for urban employees③The [medical](javascript:void(0);) [insurance](javascript:void(0);) [for](javascript:void(0);) [urban](javascript:void(0);) [residents](javascript:void(0);) ④The commercial insurance ⑤No |  |
| **A6** | Self-reported sex orientation？   1. Homosexual ②Heterosexual ③Bisexual ④Indeterminate |  |
| **A7** | Venues to seek for sexual partners:   1. Bars, dance halls, tea room, club ②Bath, sauna, foot massage, massage ③Park, public toilets, the grass ④Internet **⑤**Others（note） |  |
| **A8** | Locally, how many MSM do you know about in your circle of friends_____?**[Knowing about means you will be able to know the other face, know the name or alias, nicknames, and contact information, and can be in touch with the other in a month. Give some time to the participants for thinking carefully]** | |
| **A9** | The relationship with the male who recommended you to participate in this program:   1. The past sexual partners**（before 6 months）②**The sexual partners in the past 6 months **③**Good friends   **④**Common friends **⑤**Acquaintances **⑥**Strangers |  |
| **A10** | Self-perceived status of physical health about yourself？**①**Very well **②**Well **③**Normal **④**Not well **⑤**Very bad |  |

**B. Sexual behavioral characteristics and sexual partner’ network**

| **B1** | Age of sexual debut with a man: ___years old | | | | | | | |
| --- | --- | --- | --- | --- | --- | --- | --- | --- |
| **B2** | Number of **male** sexual partners with you with **anal intercourse** in the past **6** months________**（If there is no, jump to B4）** | | | | | | | |
| **B3** | Frequency of condom usage engaging in **anal sex** with **male** sexual partners in the past **6** months？ | | | | | | | |
|  | **B31** | Anal intercourse with regular male sexual partners：**①**Never **②**Sometimes **③**Always **④**There was no such sexual activity | | | | | |  |
|  | **B32** | Anal intercourse with casual male sexual partners：**（Neither commercial or regular）**：**①**Never **②**Sometimes **③**Always **④**There was no such sexual activity | | | | | |  |
|  | **B33** | Anal intercourse with male sex workers：**①**Never **②**Sometimes **③**Always **④**There was no such sexual activity | | | | | |  |
|  | **B34** | Group anal intercourse：**①**Never **②**Sometimes **③**Always **④**There was no such sexual activity | | | | | |  |
|  | **B35** | Anal intercourse after drinking wine or beer: **①**Never **②**Sometimes **③**Always **④**There was no such sexual activity | | | | | |  |
|  | **B36** | Anal intercourse after taking drugs：**①**Never **②**Sometimes **③**Always **④**There was no such sexual activity | | | | | |  |
| **B4** | Number of **male** sexual partners with you with **oral sex** in the past **6** months**（If there is no, jump to B6）** | | | | | | | |
| **B5** | Frequency of condom usage engaging in **oral sex** with **male** sexual partners in the past **6** months？ | | | | | | | |
|  | **B51** | Oral sex with regular male sexual partners：**①**Never **②**Sometimes **③**Always **④**There was no such sexual activity | | | | | |  |
|  | **B52** | Oral sex with casual male sexual partners：**（Neither commercial or regular）**：**①**Never **②**Sometimes **③**Always **④**There was no such sexual activity | | | | | |  |
|  | **B53** | Oral sex with male sex workers：**①**Never **②**Sometimes **③**Always **④**There was no such sexual activity | | | | | |  |
| **B6** | Please recall the male sexual partner in the recent 1 year one by one , you don't need to tell their names, but in order to help you recall this information, you can give them the number or use code (**Please according to frequency of sex from more to less listed in sequence, less than 5 people, all listed; more than 5 people, fill in only five people**) | | | | | | | |
|  | **Sexual partner’ characteristics** | | **No.1** | **No.2** | **No.3** | **No.4** | **No.5** | |
|  | **B61** | **Relationship between you and male sexual partners：**  **①** Regular male sexual partners **②**Casual male sexual partners**（Neither commercial nor regular）③** Commercial male sexual behavior  **④**Other___ |  |  |  |  |  | |
|  | **B62** | **Age of the sexual partner(years)：①**<20 **②**20～**③**30～**④**40～**⑤**≥50 |  |  |  |  |  | |
|  | **B63** | **Frequency of sexual behavior：**  **①**≥twice every week **②**4～7times every month **③**Once to 3 times every month **④**<once every month |  |  |  |  |  | |
|  | **B64** | **HIV-positive status of sexual partners：①**Positive **②**Negative **③**Unclear |  |  |  |  |  | |
| **B7** | Age of sexual debut with a woman:**①**years old **②**There was no sex with women**（Jump to C1）** | | | | | | |  |
| **B8** | Number of **female** sexual partners with you with **sex** in the past **6** months________**（If there is no, jump to C1）** | | | | | | | |
| **B9** | Frequency of condom usage engaging in **anal sex** with **female** sexual partners in the past **6** months？ | | | | | | | |
|  | **B91** | Spouse**(regular)**female sexual partners: **①**Never **②**Sometimes **③**Always **④**There was no such sexual activity | | | | | |  |
|  | **B92** | Casual female sexual partners （**Neither commercial or regular**）：**①**Never **②**Sometimes **③**Always **④**There was no such sexual activity | | | | | |  |
|  | **B93** | Female sex workers：**①**Never **②**Sometimes **③**Always **④**There was no such sexual activity | | | | | |  |

**C. Behavior and social cultural characteristics**

| **C1** | Do you smoke? **[Smoking means smoking one or more cigarettes every day for more than a year or smoking more than 300 cigarettes in the short term (3 months or less**)**]**   1. Current smoker **②**Former smoker**(Having never smoked in the past 6 months)** 2. Never smoke **(Jump to D2)** | |  |
| --- | --- | --- | --- |
|  | **C11** | When did you start to smoke？Age_____ (or _____year).  Accumulated ____years of smoking excluding time not to smoke up to now. | |
|  | **C12** | **Number of cigarettes you smoke on average every day. (Generally a pack of cigarettes is 20 cigarettes.)________** | |
| **C2** | Do you drink**[Drinking refers to drinking alcohol intake of 100 grams per week (2 liang), such as 250 grams of 40 degrees wine consumption, 1 kg of 10 degrees wine intake]**   1. Drinking now **②**Drinking before **(No drinking in the past six months) ③**Never drinking**(Jump to D5)** | |  |
|  | **C21** | From ____aged years old (or ___years), you started to smoke.  Accumulated ____years of drinking excluding time not to drink up to now. | |
|  | **C22** | Times of drinking a week on average(drinking intake drink every time generally)：   1. Beer___ bottles(___ml) **②**Red Winebottles（ml）**③**White spirit ___liang（___degree wine） 2. Yellow wine ___liang（degree wine）**⑤**Others，wine**(What kind)** ___ml（___degree wine） | |
| **C3** | If you were drunk in the past year? **①**Often **②**Sometimes **③**Never**(Jump to D5)** | |  |
| **C4** | If you want or need to reduce your drink in the past 1 year? **①**Yes **②**No | |  |
| **C5** | If you have more than amount of drug use in the past 1 year(**including Psychotropic drugs, such as sedative hypnotics, caffeine, wheat carlin, LSD, and narcotic drugs, such as opiates, cocaine, cannabinoids, etc**)?   1. Often **②**Sometimes **③**Never**(Jump to D7)** | |  |
| **C6** | If you want or need to reduce your drug use? **①**Yes **②**No | |  |
| **C7** | Have you ever suicide tendency？**①**Never **②**Sometimes **③**Often | |  |
| **C8** | If you suffered from a male sexual partner violence?（**violence includes physical, sexual or mental violence**）   1. Never **②**Sometimes **③**Often | |  |
| **C9** | If you suffered from sexual abuse in your childhood?**①**Yes **②**No | |  |
| **C10** | What impact do you think the current social perception about MSM will have on sexual behavior?   1. Increasing protected sex with a male**②**Increasing protected sex with a female **③**Increasing unprotected sex with a male **④**Increasing unprotected sex with a female **⑤**No impact | |  |
| **C11** | Self-perceived possibility of HIV infection: **①**Probable **②**Possible **③**Unlikely **④**Impossible | |  |
| **C12** | Estimate of HIV prevalence among MSM now: **①**≤5% **②**6-10% **③**11-15% **④**16-20% **⑤**≥21% | |  |
| **C13** | **Frequency of receiving HIV/STI interventions in the past year（**including giving out a condom/lubricant or publicity materials,, venereal disease inspection or treatment, AIDS/STD consulting, the training of AIDS/STD knowledge prevention, etc**）**   1. Never **②**1~2 times a year **③**2~5 times half year **④**1~3 times a month **⑤**1~4times a month | |  |

**D．Rural MSM**

| **D1** | **In the past year**，have you had homosexual behavior in the cities of Zhejiang province？  **①**Yes **②No** |  |
| --- | --- | --- |
| **D2** | **In the past year**，have you had homosexual behavior in other province？  **①**Yes **②No** |  |

**E. The history and intention of HIV testing**

| **E1** | Have you ever undertaken an HIV testing?**①**Yes **②**No**（Jump to E6）** | |  |
| --- | --- | --- | --- |
| **E2** | Times of HIV testing in the past year:__________ | | |
| **E3** | Date of the recent HIV testing: ___month ___ year | | |
| **E4** | The type of the recent HIV testing:   1. VCT (Voluntary Counseling And Testing) **②**Testing service provided by CDC **③**Blood transfusion (blood products) **④**Blood donation **⑤**Physical examination for employment   **⑥**Premarital physical examination **⑦**Hospitalized examination **⑧**Physical examination for enrollment **⑨**Other（Please specify） | |  |
| **E5** | Do you have the habit of regular HIV testing？**①**Yes **②**No**（Jump to E6）** | |  |
|  | **E51** | The reason of regular HIV testing**（Single or multiple choices）**:   1. Health Surveillance **②**Frequent high-risk sexual behaviors **③** Distrusting the regular sexual partner **④**The regular sexual partner is positive **⑤** To start a new partnership with the other **⑥**Other（Please specify） |  |
|  | **E52** | How long do you make a HIV test?（**Jump to F1 after finishing the question）**   1. 3 months **②**Half year **③**One year **④**Other（Please specify） |  |
| **E6** | Are you willing to accept regular testing for HIV? **①**Yes**（Jump to F1）②**No | |  |
|  | **E61** | If no, the reason is______（**Single or multiple choices）**   1. Don't know where to test ②It is difficult to take a test on a regular time due to work or the living habits ③Worry about the positive test results   **④**It is not convenient to go to the test place **⑤**Fear of the discrimination after their identity exposure **⑥**There is no high-risk behavior  ⑦It is very safe for regular sexual partners **⑧**Other（Please specify） |  |

**This questionnaire is now over! Thank you for your participation! Welcome to put forward your precious suggestion for the survey!**

**­**

**The following results is completed by the investigator**

| **F1** | Syphilis testing result：the first method：①Positive ②Negative |  |
| --- | --- | --- |
| **F2** | Syphilis testing result：the second method：①Positive ②Negative |  |
| **People infected with HIV before (not including positive people for HIV testing in this survey), just fill out the G6-G8.** | | |
| **F3** | At the beginning, the screening method for HIV:________ |  |
| **F4** | HIV screening result： ①Positive ②Negative**（Finishing this survey）** |  |
| **F5** | HIV confirmed result：①Positive ②Negative**（Finishing this survey）** |  |
| **F6** | Card number for outbreak： |  |
| **F7** | Viral load results for HIV testing：Date for viral load testing：___mm/dd/yyyy | |
| **F8** | CD4 result：Date for CD4 testing：___mm/dd/yyyy | |

**­­­**

**Please make sure whether you have completed the following processes:**

1. **Participant inclusion and exclusion**②**Informed consent ③Omission items reviewing after completing questionnaire**

**Investigator： Investigation Date：_______(**mm/dd/yyyy)
