# Supplementary material for: HIV risk behavior and HIV testing among rural and urban men who have sex with men in Zhejiang Province, China: A respondent-driven sampling study
Source: PLoS One. 2020 Apr 2;15(4):e0231026. doi: 10.1371/journal.pone.0231026 (PMC7117739; doi:10.1371/journal.pone.0231026)
Supplement: Attachment — Submitted filename: Response to Reviewers.docx [file pone.0231026.s003.docx]

**Response to Reviewers**

**Dear Editor:**

On behalf of my co-authors, we thank you very much for giving us an opportunity to revise our manuscript, we appreciate editor and reviewers very much for their positive and constructive comments and suggestions on our manuscript entitled “HIV risk behavior and HIV testing among rural and urban men who have sex with men in Zhejiang Province, China: a respondent-driven sampling study”. (PONE-D-19-33057). We have studied reviewer’s comments carefully and have made revision which marked in red in the paper. We have tried our best to revise our manuscript according to the comments. Attached please find the revised version, which we would like to submit for your kind consideration.

We would like to express our great appreciation to you and reviewers for comments on our paper. Looking forward to hearing from you.

Thank you and best regards.

Yours sincerely,

Lin He

Corresponding author:

Xiaohong Pan

|  | Response to Academic Editor |  |
| --- | --- | --- |
|  | Comments | Response |
| 1 | Author needs to provide the sampling principle, and why choosed this rural district and urban area for study? | Thank you  **For the sampling:**  Using respondent-driven sampling (RDS) to recruit participants, the study initially recruited eight eligible participants in rural areas and five participants in urban areas as seeds. Participants were recruited from the local MSM population based on diverse characteristics, including age and social demographics. The initial seeds were asked to recruit partners or peers to participate in the survey. After the seeds completed the study questionnaires and underwent HIV testing, they were provided with three recruitment coupons to incentivize other participants. Using these incentives, the seed participants then recruited a maximum of three MSM peers from their social network to participate in the survey. These new recruits were then asked to recruit up to three additional peers to participate in the study.  This process was repeated until equilibrium was reached. More specifically, the sample composition was considered to have reached equilibrium when their socio-demographic characteristics were stable and would not change much for further recruitment of participants (the key sociodemographic characteristics such as age, marital status, educational level, monthly income, and sexual orientation changed < 2% in a subsequent wave recruitment of peers compared with the previous one), irrespective of the characteristics of the initial seeds. After reaching equilibrium, we stop the recruit.  On the other than, we can calculate the sample size based on the use of HIV prevalence ranged from 5% to 10%; and HIV testing in past year range from 40% to 60%. P-values≤0.1 and β=0.1 were considered statistically significant, and precision=0.1. Thus, it was estimated that the minimum sample size required for this study would be 402 people. The sample size was calculated using PASS software (version 11.0). We total recruited 710 participants; the sampling size was enough.  **Why choose rural and urban**  In China, **HIV prevalence among MSM have significance difference between developed and developing areas**, in developed areas such as Beijing, Shanghai and Shenzhen, the HIV prevalence was about 8% (reference 4, 6, 17), while in developing areas such as Hebei, Guangxi and Yunnan Province, the HIV prevalence was low than 4% (reference 11, 17, 20).  We recruited two eligible rural participants as seeds who were willing to participant the survey, they were from Quzhou city, one was from Kaihua county and the other from Jiangshan city. For the urban seeds we recruited from Lucheng district, Wenzhou city.  Quzhou belong to economic developing areas, 44.3% residents are peasants, many young people have a short history of urban mobility, and there are no public meeting places for MSM, such as bathes or parks. Wenzhou located in a coastal city and economically developed area, Lucheng district is the unique downtown areas in Wenzhou city, in Lucheng district 90.5% residents are citizens, have many migrant MSM work here and have several public places for MSM such as bathes and parks.  Quzhou and Wenzhou are representative areas of rural and urban in Zhejiang Province.  We have added the sampling principle from Line 131 to 146, and why choosed the areas from Line 149 to 165. |
| 2 | How about the overall epidemic condition of MSM in Zhejiang Province and China? I can not find the necessarity of this investigation. | Zhejiang Province is located on the east coast of China, is economically developed, and has had an HIV prevalence of 8% among MSM since 2008. During this same time period, the national HIV prevalence was 4.9% between 2008-2009 (reference 17, published in 2013). The HIV epidemic in the Zhejiang Province forecasts a future trend for China.  **The reason for the necessarily of this investigation**   (1) In China and Zhejiang Province, the HIV sentinel surveillance for MSM set in urbans, and no sentinel surveillance conduct in rural areas, very little data of HIV from rural MSM. (2) In China and Zhejiang Province, the rural population has the characteristics of short-term and/or long-term migrant to the urban areas. In addition, the rural MSM gathering urban places such as bathes or parks to find sexual partners, as well as the use of MSM Group data software, may promote the interaction between the urban and rural MSM, such result in the HIV spread in rural areas. (3) In China, especially in rural areas, cultural and familial pressure drive a greater proportion of MSM to have female partners than anywhere else in the world. **The authors all from CDC system, as far as we known, the intervention and testing for HIV mainly conducted in urban areas, the rural areas can mistakenly appear to have a low MSM population density, and very little intervention conducted in rural aeras, the HIV prevalence, testing information among MSM in rural areas was unknown in Zhejiang Province** and most areas of China. If we want to control and prevention HIV epidemic, the rural areas is the key, because it was estimated that 1%-2% of men in rural areas were MSM, and more than 40% of individuals lived in rural areas in China.   So we think it is necessary for the investigation. We have added the data from Line 110to 127. |
| 3 | The writing needs to be improved substantially. | After revising the revision manuscript, we used Editage (English language service company) for performing a recheck of the revised manuscript, and improved the English language. |
| 4 | Just simple statistical analysis was performed and the research method did not support the conclusion effectively. | Our study focused on the differences between rural and urban MSMs in HIV testing and infection, ect. The differences in social demographic characteristics among rural and urban MSM were mainly used multivariate Logistic regression models to support the conclusion.  From Line 179 to 192.  For the conclusion: we deleted the high HIV infection in rural area, it was not supporting the conclusion.  For the higher high-risk behavior (in table 3), multivariate Logistic regression models showed urban MSM had high condom use compare to rural MSM (AOR=7.46, 95% CI 4.45-12.52), it meant that rural MSM had higher high-risk behaviors.  For the low HIV testing in rural MSM compared to urban MSM, in table 4, rural (41.8%) vs urban (43.6%), multivariate Logistic regression showed urban MSM had higher HIV testing rate (AOR=1.81, 95% CI 1.18-2.79), it meant rural MSM had low HIV testing rate. |

|  | Response to Reviewer #1 |  |
| --- | --- | --- |
|  | Comments | Reply |
| 1 | The manuscript is focusing on the differences between rural and urban MSMs in HIV testing and infection, ect. Despite of higher proportion of high risk behavior, there was a lower rate of HIV infection in rural MSMs, as compared to unban MSMs. The authors did not analyze the reasons for the paradox. From the description, a proportion of the study rural MSMs had homosexual behavior in cities during last year, and some unban MSMs had health insurance in rural areas. Are the two study population discrete or mixed up? | Thank you, very good comment.  During the investigation, we distinguished the rural and urban MSM based on the recent residence areas, participants residing in Kaihua and Jiangshan more than 3 months as rural MSM, and in Lucheng as urban MSM, the survey areas of rural and urban has long distance (more than 320 kilometers), the two population were discrete.  For the reason why rural MSM had higher proportion of high-risk behavior, lower rate of HIV infection compared to unban MSM.  The reason may be that: We found the rural MSM had reported high risk behavior than urban MSM, yet paradoxically had a lower rate of HIV infection than unban MSM. In China, especially in rural areas, cultural and familial pressures tend to make MSM unwilling to disclosure their sexual orientation. Thus, MSM tend to prefer to gather in the urban areas which are more inclusive and accepting of MSM. The HIV epidemic among MSM in China is concentrated mainly in urban cities. Our study showed that some MSM living in rural areas had engaged in sex with men in cities during the previous year, and that some unban MSM had health insurance in rural areas. The above results indicated that rural MSM had a lower prevalence of HIV infection because the HIV epidemic had longer time in urban areas, resulted in urbans areas had more HIV-infected MSM and high HIV prevalence, while the HIV epidemic had shorter time in rural areas, had little HIV-infected MSM and low HIV prevalence. However, with the higher high-risk behavior and more HIV-infected MSM rural areas, there is a potential for a rapid increase in the incidence of HIV infection in the rural population. Additional intervention measures should immediately be implemented among rural MSM population to reduce HIV transmission.  We have added the reasons for the paradox from Line 331 to 346. |
